# Antibodies Isolated from Rheumatoid Arthritis Patients against Lysine-Containing *Proteus mirabilis* O3 (S1959) Lipopolysaccharide May React with Collagen Type I

**DOI:** 10.3390/ijms21249635

**Published:** 2020-12-17

**Authors:** Katarzyna Durlik-Popińska, Paulina Żarnowiec, Łukasz Lechowicz, Józef Gawęda, Wiesław Kaca

**Affiliations:** 1 Department of Microbiology and Parasitology, Institute of Biology, Jan Kochanowski University, 25-369 Kielce, Poland; paulina.zarnowiec@ujk.edu.pl (P.Ż.); lukasz.lechowicz@ujk.edu.pl (Ł.L.); wieslaw.kaca@ujk.edu.pl (W.K.); 2 Rheumatology Clinic ARTIMED, 25-022 Kielce, Poland; jozef.gaweda@interia.eu

**Keywords:** rheumatic diseases, rheumatoid arthritis, *Proteus mirabilis*, lipopolysaccharide, rheumatoid factor, anti-citrullinated protein antibodies

## Abstract

Most rheumatic diseases, including rheumatoid arthritis (RA), are characterized by immune disorders that affect antibody activity. In the present study, using Dot blot and ELISA assay, we showed that patients with rheumatic disease produced significantly more antibodies against lipopolysaccharide (LPS) *P. mirabilis* O3 compared to healthy donors (*p* < 0.05), and affinity purified antibodies against LPS O3 may cross-react with collagen type I. It was demonstrated that purified of antibodies isolated from RA patients sera, reacted stronger with the collagen than healthy donors (*p* = 0.015), and cross-reaction was correlated with level of anti-citrullinated peptide antibodies (r = 0.7, *p* = 0.003). Moreover, using six different lipopolysaccharides were demonstrated the significant correlations in sera reactivity among lysine-containing lipopolysaccharides observed in patients’ sera (*p* < 0.05). Using Attenuated Total Reflection Fourier Transform Infrared Spectroscopy (ATR-FTIR) it was shown that unique wavenumbers of sera spectra correlate with reactivity with lipopolysaccharides allowing distinguish patients from healthy blood donors. Antibodies adsorption by synthetic antigens shows that in patients’ group anti-LPS O3 antibodies can be adsorbed by both amides of galacturonic acid and lysine or threonine, which suggests less specificity of antibodies binding with non-carbohydrate LPS component. The observed correlations suggest that non-carbohydrate components of LPS may be an important epitope for less specific anti-LPS antibodies, which might lead to cross-reactions and affect disease development.

## 1. Introduction

Rheumatic diseases are a family of approximately 100 chronic illnesses characterized by inflammation that leads to damage and loss of function in the joints, tendons, ligaments, bones, and muscles. One of the most common rheumatic diseases is rheumatoid arthritis (RA) [1]. RA is the most common autoimmune, inflammatory disease and affects 0.5–1% of the adult population worldwide. There are 20–50 cases per 100,000 annually, most commonly in women aged >40 years [2]. RA mainly attacks small joints (hands and feet); it is characterized by hyperplasia of the synovial membrane, increase in macrophages, high levels of proinflammatory cytokines, activation of catalytic enzymes responsible for degradation of extracellular matrix, as well as the expression of autoantibodies, such as rheumatoid factor (RF) or anti-citrullinated protein antibodies (ACPAs). RA causes the degradation of articular and periarticular tissues, distortion and impaired function of the tissues, and consequently, permanent disability [3]. The etiological basis of RA is not fully understood. It is believed that RA development is dependent on genetic factors, HLA polymorphisms, and environmental factors such as smoking and infectious agents [4]. It is assumed that autoimmunity caused by infective agents is induced by molecular mimicry, cross-reactions, or the formation of immunological complexes [5]. One of the most common bacteria linked with the etiopathogenesis and development of RA is *Proteus mirabilis*. The evidence for this can be briefly summarized as follows: (1) *P. mirabilis* is isolated more frequently from biological samples of RA patients, compared to healthy controls; (2) serum, blood and urine of RA patients contain higher levels of antibodies to *P. mirabilis*; (3) molecular mimicry based on amino acid sequence similarity has been found between hemolysin HpmA (ESRRAL) and the RA molecular marker, HLA-DR4 (EQRRAA); (4) rabbits produce anti-*Proteus* antibodies after vaccination with DR4 cells; (5) the *Proteus* urease amino acid sequence IRRET has homology with the LRREI motif that is present in type XI collagen; and (6) immunological cross-reactivity occurs between *Proteus* and host peptides [6,7,8,9,10,11].

*P. mirabilis*, like other Gram-negative bacteria, produces lipopolysaccharide (LPS) on its surface [2,12]. The proinflammatory properties of LPS make it an important factor in RA development [2,13,14,15]. Moreover, LPS is an important immunogen [16]. *P. mirabilis* has approximately 80 serogroups based on the chemical structure of the O antigen, which is characterized by “decoration” of the non-carbohydrate component of the carbohydrate chain, including phosphocholine, alcohols or amino acids [17,18]. Most isolates from clinical samples belong to serogroup O3 and contain galacturonic acid amidated with lysine [19]. Animal studies show that amid of lysine and galacturonic acid (Lys-GalA) is an important epitope which contributes to cross-reaction between different serogroups of *P. mirabilis* [20,21]. It has been previously shown that human sera have antibodies against LPS *P. mirabilis* S1959, O3 (anti-LPS O3) and a small fraction of antibodies against the synthetic antigen, Lys-GalA. Moreover, it have been shown that antibodies against Lys-GalA can be partially adsorbed by collagen I. This suggests that antibodies against *P. mirabilis* LPS might cross-react with human tissue [22]. Studies based on the detection and description of neo-epitopes in arthritis show that enzymatic degradation of joint collagen, including collagen I, may affect biomarkers production useful in diagnostics indicates a role of collagen type I in disease development [23]. Collagen I has an exposes lysine residue at position 337 that is necessary to stabilize the correct protein structure and binding with low-density lipoprotein receptors [24]. Moreover, hydroxylysine present in structure of collagen type I is glycosylated by galactose and glucose [25], which may affect antigenicity the tissue. This suggests that antibodies against *P. mirabilis* LPS might cross-react with human tissue and affect disease development. The aims of the present study were to detect antibodies against lysine-containing lipopolysaccharides of *P. mirabilis* and its synthetic oligosaccharide fragments in the sera of patients with rheumatic diseases, and to correlate the serological response between LPSs and rheumatic and inflammation markers [RF, ACPA, C- reactive protein (CRP), and erythrocyte sedimentation rate (ESR)]. Moreover, to purify anti-LPS antibodies and conduct reaction with collagen type I.

## 2. Results

### 2.1. Detection of Antibodies Binding to Lipopolysaccharides Containing Lysine in Carbohydrate Chains

**Figure 1:**
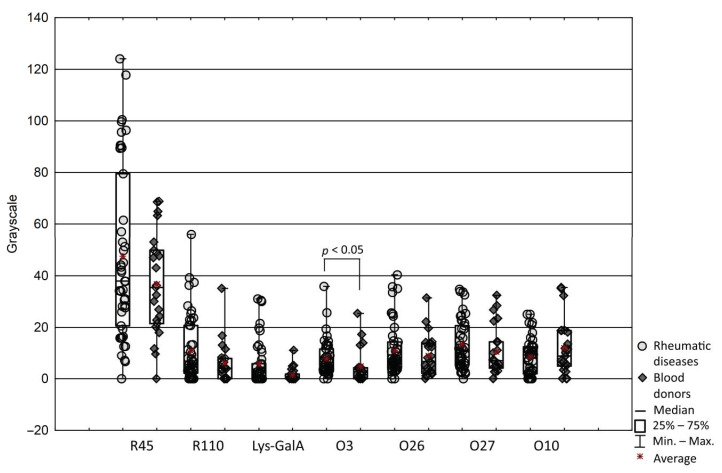
Reactions of sera samples with lipopolysaccharides and Lys_galA measured by gray scale.

Firstly, to characterize the antibody responses to lysine- containing lipopolysaccharides of *P. mirabilis*, the possible effects of aging, gender and treatment on sera reaction were examined. The reaction of sera with LPS O3 and other antigens containing lysine residues, such as O26, and O27 LPSs containing lysine in the O antigen, and O10 LPS containing lysine residue in the core part were measured. For better interpretation of the obtained results, reactions with LPS R45 and R110 lacking the lysine epitope, and synthetic oligosaccharide fragment Lys-GalA were performed. To detect serum antibodies against LPSs, a dot blot assay was used. Based on observations it was considered that the antibody responses to tested LPSs are unique to individuals and generally remain the same level in both of groups. Unexpectedly, the most reactive LPS was R45, consisting of lipid A and Kdo region and the less reactive compound was synthetic Lys-GalA. Antibodies reacted stronger with R45 LPS compared to other types of LPS in most of the tested sera (*p* = 0.0001). Although there were differences between the reactions of patients and healthy donors, patients’ sera were significantly more reactive only with LPS O3 compared to sera from healthy donors (*p* = 0.016) (Figure 1). No apparent correlation was observed between reaction with any LPS and age in both groups, except in the patients’ sera reaction with LPS R45 (r = 0.32, *p* < 0.05). Significant correlation was observed between gender and patients’ sera reaction with Lys-GalA (r = 0.35, *p* = 0.004), indicating that male patients had an increased reaction. Among RA patients, 14 individuals were treated with methotrexate (MTX). Interestingly, a negative correlation was observed between MTX treatment and reaction with Lys-GalA (r = −0.44, *p* = 0.02) and LPS O27 (r = −0.4, *p* = 0.04), indicating the possibility that MTX treatment may decrease the antibodies reaction, suggesting a positive result of the treatment.

**Figure 2:**
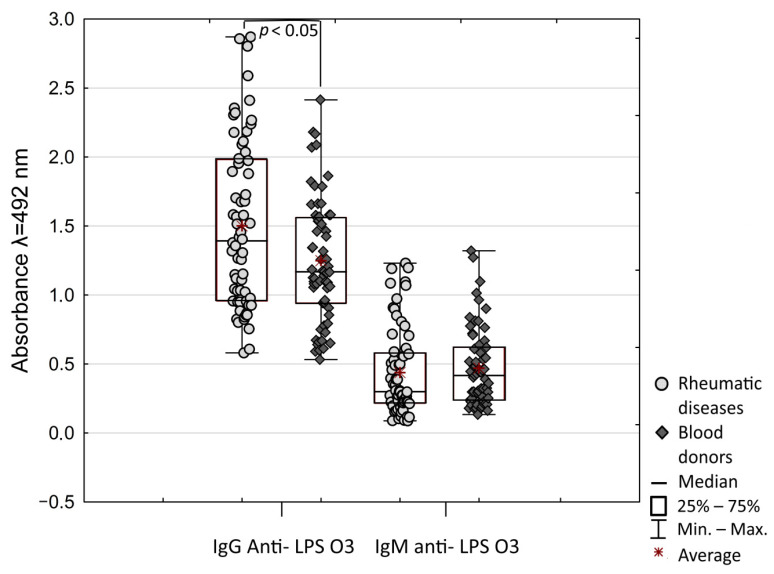
Absorbance of IgG and IgM class antibodies reactions with LPS O3.

To confirm the increased reactivity of patients’ sera with LPS O3, ELISA assay anti- LPS O3 IgG and IgM antibody reaction was conducted. As with the Dot blot, compared to healthy donors, patients’ sera were more reactive with LPS O3 (Figure 2). The difference was observed only for IgG (*p* = 0.041). IgG antibody reaction was significant higher compared to IgM in both groups (*p* < 0.001). Despite the slightly higher reaction of anti-LPS O3 IgM in the healthy donors’ group, there were no significant differences in antibodies reaction between patients and healthy donors. Although the value IgM/IgG ratio was more scattered in patient individuals, healthy donors had a significantly higher IgM/IgG ratio compared to rheumatic disease patients (*p* = 0.003). The median (IQR) of IgM/IgG value was 0.26 (0.17–0.33) in the patients group compared to 0.33 (0.24–0.53) in healthy donors. The high reaction of IgG and low reaction of IgM class antibodies suggest that patients may have been exposed on *P. mirabilis* O3 infections in the past. Anti- LPS O3 IgG and IgM antibodies significantly positively correlated in the patients group (r = 0.48, *p* < 0.0001) and healthy donors (r = 0.47, *p* < 0.0001). Moreover, a significant negative correlation is observed between age and IgM reaction (r = −0.51, *p* < 0.0001), and between age and IgG reaction (r = −0.38, *p* = 0.003) in the patients group and not observed in healthy donors. Similarly, the IgM/IgG ratio was significantly negatively correlated with age (r = −0.35, *p* = 0.006). Importantly, correlation was observed between anti-LPS O3 IgG decreased reaction and MTX treatment (r = −0.38, *p* = 0.002); it was not observed in results based on the Dot blot assay, but confirmed the possibility that MTX treatment may reduce antibodies response.

### 2.2. Correlation of Sera Reaction to Synthetic Lys-GalA and with LPS Containing Lysine Residues in the O antigen

**Figure 3:**
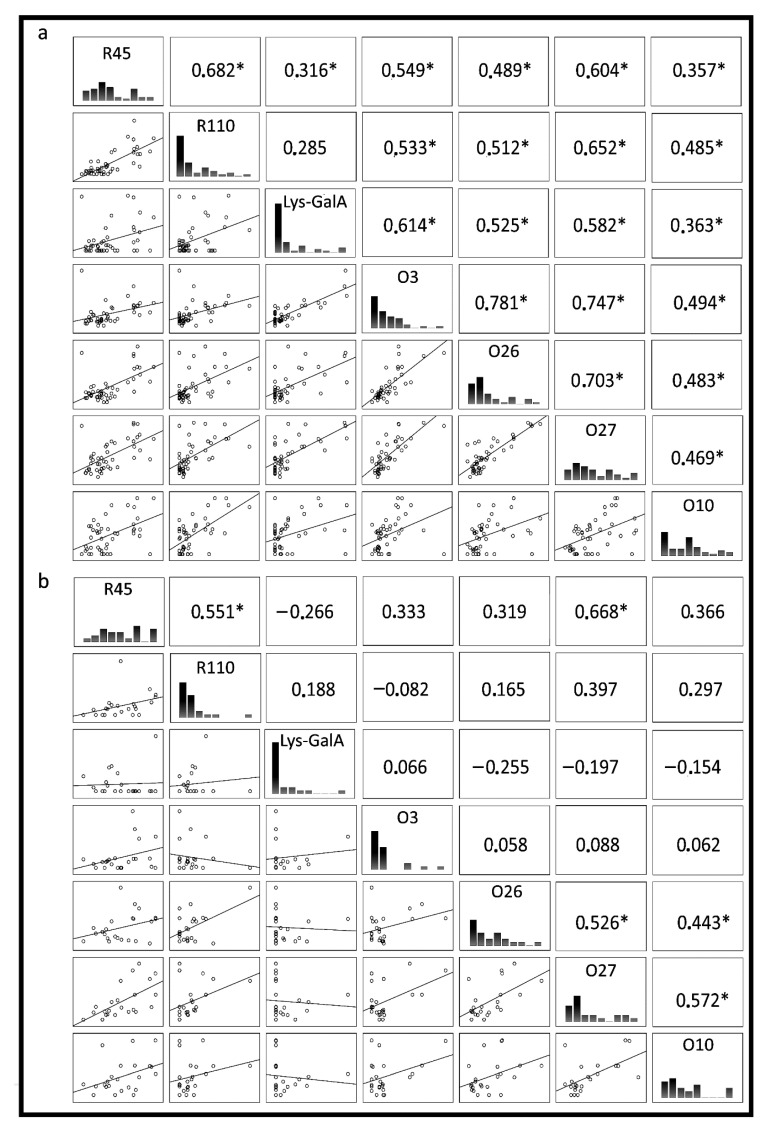
Spearman correlation between: (**a**) rheumatic disease patients‘ sera, (**b**) healthy blood donors’ sera, reacting with *P. mirabilis* antigens: smooth LPSs: O3, O26, O27, O10; mutant LPS O3s: R110 and R45 and synthetic oligosaccharide fragment Lys-GalA. The linear relationship was determined by Spearman’s correlation coefficient (bottom left corner), histogram of serum reactivity (middle) and Spearman rank correlation of 1 or −1 (upper right corner). * Indicates a significant correlation.

To further characterize the antibody responses to lysine-containing lipopolysaccharides of *P. mirabilis*, the relationship between antibody responses to individual LPS was analyzed. However, no difference between patients’ and healthy donors’ sera for reactivity with most tested LPSs was observed. There were significant correlations between reactivity to LPSs O3, and O26 (r = 0.78, *p* < 0.0001) and O27 (r = 0.75, *p* < 0.0001), and between O26 and O27 (r = 0.7, *p* < 0.0001) for patients’ sera and r = 0.53 for healthy donors’ sera (*p* < 0.05) (Figure 3). In a few samples of both groups of sera, we detected a small fraction of antibodies anti-Lys-GalA (Figure 1). Despite there being differences between the reactions of patients’ and healthy donors’ sera, they were not significant. The reaction of sera with synthetic antigen was significantly correlated to reactions with LPS O3 (r = 0.61, *p* < 0.0001), O26 (r = 0.53, *p* = 0.0003) and O27 (r = 0.58, *p* < 0.0001), but only for patients’ sera (Figure 2). Moreover, correlation analyses of serum reactivity for the mutants, R45 and R110 LPS showed a significant correlation in both tested groups: in patients (r = 0.68, *p* < 0.0001) and in healthy blood donors (r = 0.51, *p* = 0.001), indicating common epitopes in the two LPS samples. There was a significant correlation between LPS R45 and all tested antigens in patients’ group of sera *p* < 0.05, and a high correlation was presented between LPS R45 and LPS O27 (r = 0.67, *p* = 0.0005). Presented correlations may be an effect of the conservative character of Lipid A exposen in LPS R45.

### 2.3. Correlation of Sera Reactivity with Markers of Rheumatoid Arthritis

Since *P. mirabilis* is linked with rheumatic arthritis development, measured the correlations among the detected rheumatoid markers RF and ACPA, and the reaction of sera with LPS were analyzed. From 67 tested serum samples, 24 were RF positive (≥30 IU/mL) and 19 were from RA patients. Most of the tested serum samples were ACPA positive (>10 IU/mL). RA patients had significantly more ACPA than healthy donors (*p* < 0.001), and significantly more RF and ACPA, compared to patients with other rheumatic diseases (*p* < 0.025). There was a significant correlation between RF and ACPA concentration in the tested sera (r = 0.59, *p* < 0.001). Moreover, there was also a significant correlation between CRP and RF (r = 0.54, *p* < 0.05), CRP and ACPA (r = 0.67, *p* < 0.05), and CRP and ESR in RA patients’ sera (r = 0.51, *p* < 0.05). Because differences in the presence of rheumatoid markers were observed, sera were divided into three groups: RA, other rheumatic diseases, and healthy donors. For RA patients, there was a significant correlation between the reaction of antibodies binding Lys-GalA and RF (r = 0.413, *p* = 0.04) and ACPA (r = 0.41, *p* = 0.04). Based on results using ELISA assay, a significant correlation was observed between ACPA and anti-LPS O3 IgM/IgG ratio in RA patients’ sera (r = 0.35, *p* = 0.004).

### 2.4. Analysis of Sera IR Spectra and Correlation with Reactivity to Lysine-Containing LPSs

**Figure 4:**
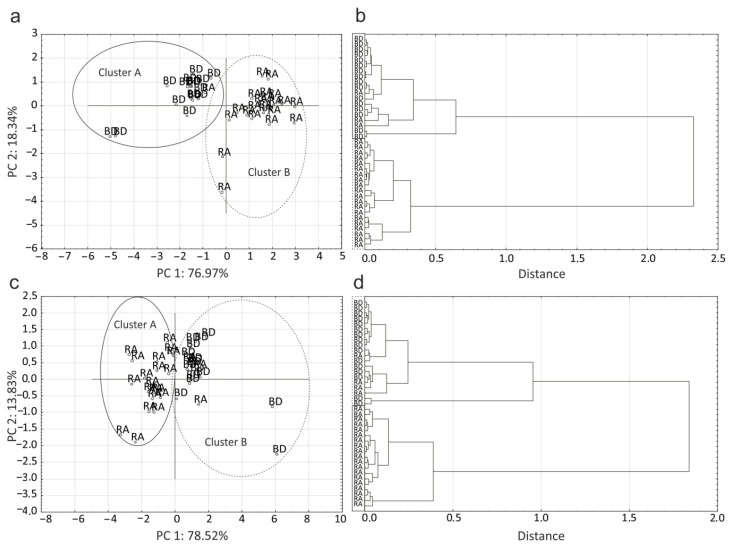
Serum clustering based on sera reactions with LPS O3 and correlated wavenumbers 1492 cm^−1^, 1493 cm^−1^, 1594 cm^−1^, 1650 cm^−1^ and 752 cm^−1^: (**a**) Principal component analysis, (**b**) Hierarchical cluster analysis. Serum clustering based on sera reactions with Lys-GalA and correlated wavenumbers 1503 cm^−1^, 1505 cm^−1^, 1505 cm^−1^, 1513 cm^−1^ and 781 cm^−1^: (**c**) Principal component analysis, (**d**) Hierarchical cluster analysis.

Due to the diversity and unique antibody responses to patients and healthy blood donor individuals. There compared healthy donors’ and RA patients’ sera by ATR FT-IR (Attenuated Total Reflection Fourier Transform Infrared Spectroscopy) and correlated their spectra with a reaction to LPSs. The two wavenumbers that strongly correlated with the sera reaction to LPS R45 were 1684 cm^−1^ located in W2 window associated with proteins and peptides, and 1318 cm^−1^ located in mixed region W3. Similar the two wavenumbers that strongly correlated with the sera reaction to LPS R110 were 1417 cm^−1^ and 1632 cm^−1^ located in W3 and W2, respectively. The sera reactions with lysine- containing lipopolysaccharides were correlated with more various wavenumbers: 1767 cm^−1^ and 976 cm^−1^ for LPS O26; 853 cm^−1^, 1434 cm^−1^, 978 cm^−1^ and 1426 cm^−1^ for LPS O27; 1535 cm^−1^, 780 cm^−1^, 801 cm^−1^, 1517 cm^−1^, 1442 cm^−1^, 781 cm^−1^, 2803 cm^−1^, 802 cm^−1^, and 494 cm^−1^ for LPS O10. The most similar wavenumbers were correlated with reactions to synthetic antigens Lys-GalA: 1503 cm^−1^, 1505 cm^−1^, 1505 cm^−1^, 1513 cm^−1^ and 781 cm^−1^ (Figure 4c,d), and LPS O3: 1492 cm^−1^, 1493 cm^−1^, 1594 cm^−1^, 1650 cm^−1^ and 752 cm^−1^ (Figure 4a,b). Since the described wavenumbers correlated with sera reactions to LPS, it was possible to use IR spectroscopy to divide the sera into two groups by using HCA (Hierarchical Cluster Analysis). Figure 4b,d show that sera were divided into groups of patients’ and healthy blood donors’ sera. Clusters of patients’ sera were homogenous and consistent, while clusters of healthy blood donors’ sera were more heterogeneous, and there were few patients’ sera in addition to healthy blood donors. Similar results were obtained by PCA (Principal Component Analysis) (Figure 4a,c).

### 2.5. Adsorption of Antibodies to Lysine-Containing LPS P. mirabilis O3 by Synthetic Antigens

**Figure 5:**
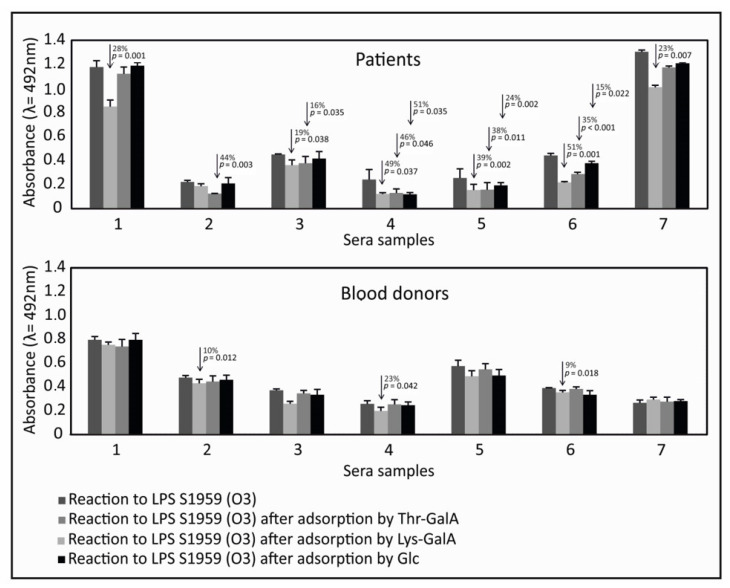
Adsorption of antibodies to *P. mirabilis* S1959 LPS O3 by synthetic antigens.

To prove the role of lysine as an epitope fragment for binding to antibodies against LPS, we selected 14 sera samples that were most reactive with Lys-GalA from patients and healthy donors (7 from rheumatic diseases and 7 from healthy blood donors). The seven most reactive samples from the patients group belonged to RA patients. Sera were adsorbed by by synthetic antigens Lys-GalA, amide of galacturonic acid and threonine (Thr-GalA), and glucose (Glc). (Figure 5). After adsorption, sera were examined for the detection of antibodies against *P. mirabilis* S1959 LPS O3 by ELISA. After background subtraction, reactions of sera against LPS O3 were low, but present. The synthetic antigen absorbed a common fraction of antibodies to Lys-GalA and *P. mirabilis* S1959 LPS O3. The amount of antibody adsorbed by Lys-GalA varied between individual samples from 0 to 49%. Synthetic antigen Lys-GalA adsorbed significantly more antibodies in patients’ sera compared to healthy donors’ sera (*p* < 0.05). Synthetic antigen Thr-GalA adsorbed similar amounts of antibodies as Lys-GalA in most of the patients’ sera. Adsorption by glucose was observed, which suggested that glucose in the side chain of LPS O3 was also an epitope for anti-LPS O3 antibodies.

### 2.6. Reaction of Affinity Purified Antibodies Against O3 with Collagen Type I

**Figure 6:**
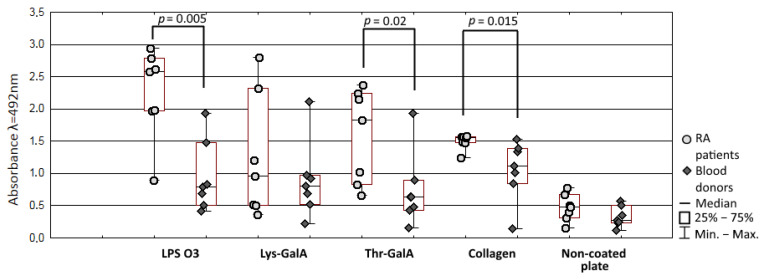
Reaction of affinity purified antibodiesagainst LPS O3 with collagen type I, LPS O3 and sytntetic fragments.

To confirm the theory that antibodies against LPS O3 may cross-react with collagen type I, there purified antibodies against LPS O3 from sera used in adsorption assay and conducted reaction with the collagen. Absorbance (λ = 280 nm) of purified antibodies samples from RA patients’ sera were significant higher compared to healthy donors (*p* = 0.007), but reactions with LPS O3, synthetic Lys-GalA and Thr-GalA, and collagen were prerformed with dilluted samples. There were no significant differences between the concentration of purified antibodies (*p* = 0.063) in RA and the healthy blood donors group, but the absorbance of the reaction was correlated with the concentration of purified antibodies samples (LPS O3 r = 0.96; collagen type I r = 0.85; Lys-GalA = 0.56; Thr-GalA = 0.92, *p* < 0.05). Rheumatoid arthritis patients anti- O3 LPS antibodies samples reacted more strongly with LPS O3 (*p* = 0.005), and collagen type I (*p* = 0.015) (Figure 6). Interestingly, there was no difference in the binding of purified antibodies with Lys-GalA between patients and healthy blood donors (*p* = 0.7) but with the Thr-GalA reaction of antibodies from patients it was increased (*p* = 0.02). There was a significant correlation between the anti-LPS O3 antibodies reaction with collagen type I and LPS O3 (r = 0.81; *p* < 0.001); Lys-GalA (r = 0.56; *p* = 0.038) and Thr-GalA (r = 0.91, *p* < 0.001). Moreover, an increased amount of purified antibodies corresponded to an increased reaction with antigen non-coated polystyrene plate, and the non-specific reaction was correlated with collagen type I (r = 0.91; *p* < 0.001), LPS O3 (r = 0.89; *p* < 0.001) Lys-GalA (r = 0.67; *p* = 0.009) and Thr-GalA (r = 0.96; *p* < 0.001), indicating that purified antibodies contain fractions of low specific characteristics for autoimmune diseases. 

## 3. Discussion

Immune system disorders resulting in the production of autoantibodies are characteristic for many rheumatic diseases. It is believed that the host produces antibodies against bacterial antigens that cross-react with human tissue [26]. One of the most varied bacterial molecules that may be a reservoir of numerous epitopes is LPS [13]. These studies are focused on the LPS of *P. mirabilis,* the bacteria linked to RA development [6,9,10]. Previous studies showed a significant difference in the level of antibodies to lysine-containing LPS O3 between RA patients and healthy donors [26]. Moreover, it has been shown that human sera produce antibodies against the amide of galacturonic acid and lysine present in the polysaccharide chain of LPS O3, and it is possible that antibodies against Lys-GalA react with collagen I [22]. Lysine is the most common amino acid incorporated in the polysaccharide chain of *P. mirabilis* LPS, and it is an important epitope responsible for cross-reactions between serogroups of bacterial LPS which confirms the results of animal studies [12,20,21]. However, to the best of the author’s knowledge, human sera reactions with other lysine-containing LPS have not been studied. Therefore, in the present study, the reactions of rheumatic disease patients’ sera with lysine-containing lipopolysaccharides *P. mirabilis* and synthetic fragment Lys-GalA were analyzed and compared. Reactions of sera were individual. There were no significant differences between almost all tested LPS, except for the LPS of the O3 serogroup confirming the earlier research [27]. Interestingly, differences were observed in reactivity of the IgG, but not IgM, class antibodies. It is worth considering whether the difference in the amount of anti-LPS antibodies is not the result of differences in the age of the studied groups. Unexpectedly, anti-LPS O3 IgM reaction appeared to be increased in healthy donors compared to the patients group, although this tendency was not significant. These observations not confirmed earlier studies about *P. mirabilis* in RA, that showed an increased level of reactions IgM antibodies against the bacteria in patients’ sera [8]. The differences may be explained by the carbohydrate structure of LPS, and differences in antibodies production. On the other hand, the lack of correlation between age and IgG antibodies reaction did not confirm previous studies showing that the amount of antibodies against LPS O3 increases with age [22]. 

Healthy donors had an increased value of IgM/IgG ratio compared to patients. The IgM/IgG ratio may be used to indicate primary infections [28]. Despite the higher value of IgM/IgG in healthy donors group of sera, these values are too low to be considered a result of infection. The low IgM/IgG value in patients group and increased IgG antibody reaction compared to healthy donors may suggest that patients had been infected with *P. mirabilis* O3 LPS.

LPS R45 showed the highest level of antibody binding. This contrasts with our previous finding that R45 bound the least antibodies in human plasma [22]. The difference can be explained by the surface to which the antigen was attached. ELISA uses polystyrene plates that bind more-hydrophilic compounds, and the dot blot assay uses PVDF membranes that bind more-hydrophobic compounds, such as lipid A. The role of antigen type in adherence to the surface confirms previous studies in which the LPSs of mutants R45 and R110 were more reactive compared to other smooth LPSs [27]. Correlations of sera reactivity between LPS R45 and other LPSs can be explained by the chemical structure of LPS. Lipopolysaccharides of *P. mirabilis* expressing the smooth type of LPS contain conservative lipid A, less conservative core and highly varied O-antigen. Each part of the LPSs carries on multiple epitopes that can be bind by the antibodies. *Proteus mirabilis* R45 and *P. mirabilis* R110 are mutants of LPS *P. mirabilis* S1959 (O3) and lack O-antigen core and O-antigen, respectively, as was described in the manuscript. That is why correlations between LPS R110, O3 and synthetic antigen are expected. Due to highly conservative character of lipid A, high reaction with LPS R45 and correlations with other LPSs may be a result of cross reaction antibodies against different LPSs of Gram-negative bacteria present in the organism. On the other hand, increased levels of antibodies against LPS O3 may be an affect of the increased level of antibodies against its lipid A cross reacting with the lipid A of other lysine-containing LPSs of *P. mirabilis*. The lack of correlation in the healthy blood donors group can be explained by the smaller number of sera, or decreased amount of antibodies to LPS O3 or less specific antibodies against lipid A in the patients’ group of sera. It has been shown that anti-lipid A antibodies may cross-react with the lipid A of different species of bacteria, as well as with molecules present in humans, such as ssDNA, cardiolipin, or ligands on human B lymphocytes. It is believed that the cross-reactivity between lipid A and other host molecules may affect the development of autoimmune diseases [29,30,31]. 

The reaction of sera with the synthetic antigen was the lowest compared to all LPS, which suggests that antibodies to the non-carbohydrate component present a small fraction in both group of sera compared to animal models. The observations described above indicate a possibility that there are more important epitopes in the LPS O3 that contribute to the reaction of patients’ sera. Even though there was no significant difference between the studied groups, statistical analysis showed a correlation between the reactivity of patients’ sera with LPS containing lysine. The correlation can be explained by the cross-reaction of antibodies to LPS O3 (the concentration of which was higher compared to that in healthy blood donors), and the important role of Lys-GalA as a cross-reactive epitope also for humans. Moreover, reaction with Lys-GalA was positively correlated with RF and ACPA. The correlations present mostly in patients’ sera may have resulted from changes in the immune response of patients and healthy donors, the specificity of the produced antibodies, and exposure to bacterial antigens. 

Due to the difference between reactivity of patients’, and healthy donors’ sera, it has been decided to compare their IR spectra with LPS reactions. Using mathematical methods, wavenumbers were found and correlated with sera reactions. These correlations were used to distinguish sera into two groups. In addition, a similarity between wavenumbers has been observed correlated to reactions of LPS O3 and synthetic Lys-GalA, which may indicate the similar underlying reactions between both of the antigens (e.g., similar fraction of antibodies binding to common epitope). Moreover, wavenumbers from 1492 cm^−1^ to 1505 cm^−1^, and 752 cm^−1^ to 781 cm^−1^ similar between LPS O3 and Lys-GalA, correspond to aromatic amino acid residue and aromatic bounds in finger printing regions, respectively. Division based on correlation between reaction to LPS and wavenumbers resulted in heterogeneous groups dominated by patients or healthy blood donors. The presence of single individuals in a given group may confirm the lack of statistical differences between the groups, although patients’ sera reactivity with LPS appeared to be increased compared to healthy donors. This could be due to many factors, such as age, genetic predisposition, antigen exposure or treatment. An example which may be used is the above observation that, in patients treated MTX, reaction with Lys-GalA and O3 LPS was lower. Results obtained with the correlation of MTX treatment and sera reactivity do not confirm earlier studies that show that MTX treatment patients had increased reaction with LPS of environment bacteria [5]. 

It is believed that *P. mirabilis* infection is a risk factor for the development of RA; therefore, the correlation between RA markers and the response of patients’ sera to *P. mirabilis* LPSs was analyzed. Only synthetic Lys-GalA was significantly correlated with RA markers, which suggests that disorders of the immune system may affect production small fractions of diverse, less-specific antibodies against bacterial components which may be associated with RA development. To prove the importance of lysine in the binding of anti-LPS *P. mirabilis* antibodies, antibodies to LPS O3 were adsorbed by the synthetic antigens Lys-GalA, Thr-GalA, and Glc. Both, Lys-GalA and Thr-GalA adsorbed similar amount of antibodies to LPS O3. It showed that human antibodies binding to Lys-GalA may be less specific compared to animal antibodies [32], which may affect their cross-reactivity with human tissue. The most important finding in this study is that antibodies against lipopolysaccharide may react with human tissues such as collagen type I. It is possible that the reaction may be an effect of the cross reaction of less specific antibodies against amide Lys-GalA present in the O-antigen with glycosylated lysine in the collagen. Glycosylated hydroxylysine (Lys-O-Gal and Lys-O-GalGlc) is a unique and specific post-translational collagen modification that is present in type I collagen. Lys-O-Gal is found predominantly in bone tissue, and Lys-O-GalGlc is found predominantly in the skin. Similar to type II collagen, type I collagen is also present in joints as a component of bones and soft tissue surrounding the joint. That cross reaction between anti-LPS O3 antibodies may affect disease development. Moreover, glycosylated lysine is also present in collagen type II; therefore, it is not out of the question that anti-LPS antibodies reacting with type I collagen may also react with type II collagen and play a role in epitope spreading [23,25,33,34]. Significant correlations between the concentration of antibodies and reactions with lipopolysaccharide *P. mirabilis* O3 confirm the possibility that the reaction with collagen is a result of the cross-reaction of purified antibodies. Unexpectedly, the reaction of purified antibodies with synthetic fragment Lys-GalA was not increased in patients’ sera. However, correlation between Lys-GalA, LPS O3 and collagen type I is observed. Interestingly, patients’ antibodies reaction with Thr-GalA was significant higher compared to healthy donors. Taking results obtained using adsorption assay and high reaction purified anti-LPS O3 antibodies with Thr-GalA together, it is suggested that some fraction of antibodies binding with Lys-GalA epitope of LPS O3 is less specific, and may be in response to the reaction with collagen. The cross-reaction of antibodies against LPS O3 with collagen is present in the patients and healthy donors group of sera, but the positive correlation between the level of ACPAs and collagen binding by purified antibodies suggests a putative relation with RA development. However, the current findings do not prove that purified antibodies have a pathogenic effect. Therefore, further studies are required to clarify the role of cross reactive antibodies to LPS *P. mirabilis* O3 in immune system disorder development.

## 4. Materials and Methods 

### 4.1. Lipopolysaccharides

LPSs extracted by phenol-water method from *P. mirabilis* strains S1959 (O3), PrK49/57 (O26), 50/57 (O27) contained lysine in O antigen repeating unit, and LPS of *P. mirabilis* HI 4320 (O10) contained lysine in the core region. LPSs extracted using the PCP method from Ra mutant of *P. mirabilis* S1959 (R110) had no O polysaccharide part, and the Re mutant of *P. mirabilis* 1959 (R45) had only a fragment of the lipid A and two rests of Kdo [22,35,36].

### 4.2. Synthetic Antigens

Synthetic 2-acrylamidoethyl α-glycoside of Lys-GalA, Thr-GalA and Glc, representing a partial structure of the *P. mirabilis* lipopolysaccharide, was synthetized as described previously [37].

### 4.3. Sera

Sera from 44 patients (mean age 55.9 ± 14.2 years, 9 male and 35 female) with rheumatic diseases were obtained from Swietokrzyskie Rheumatology Center, Konskie, Poland. The rheumatic diseases were: 26 (59.1%,) samples of RA sera, 1 (2.3%) juvenile idiopathic arthritis, 1 (2.3%) systemic vasculitis sample, 7 (16%) osteoarthritis samples, 4 (9%) ankylosing spondylitis samples, 1 (2.3%) sample lupus erythematosus, 2 (4.5%) reactive arthritis, and 2 (4.5%) undifferentiated spondylarthritis. Sera from 23 healthy blood donors without known rheumatic disease (mean age 44.7 ± 5.2 years, 2 male and 20 female) were obtained from Swietokrzyskie Blood Center, Kielce, Poland. Patients’ sera levels of inflammatory markers: C-reactive protein (CRP) and erythrocyte sedimentation rate (ESR) were 0.75–85.9 mg/mL and 4–83 mm/h, respectively. Samples were collected with the approval of the Ethics Committee of the Regional Chamber of Physicians in Kielce No. 30/2019-VII, 17.10.2019 r.

### 4.4. Dot Blot Assay

The dot blot assay was carried out with a panel of *P. mirabilis* LPS and the synthetic antigen, Lys-GalA. LPSs and Lys-GalA (10 µg/mL) were dotted on a polyvinylidene difluoride (PVDF) (Merck, Darmstadt, Germany) membrane and air dried. The membranes were washed three times Tris-buffered saline (TBS), blocked with 2% bovine serum albumin, BSA (Sigma-Aldrich) in TBS at 37 °C for 1 h, and then washed again three times with TBS. The membrane was incubated with patient sera diluted 1:1000 (*v*/*v*) in 1% BSA TBS for 1 h at 37 °C. The membranes were washed three times with TBS. Next, the membrane was incubated with horseradish-peroxidase-conjugated anti-human IgG (Sigma-Aldrich, St. Louis, MO, USA) diluted 1:1000 (*v*/*v*) at 37 °C for 1 h and washed with TBS. The membrane was further developed with the chromogenic substrate, 4-chloro-1-naphthol (Sigma-Aldrich, USA) and H2O2 (Sigma-Aldrich). Reaction with rabbit sera anti-LPS O3 was used as positive control. Drops of solvent without antigen were used as negative controls. The PVDF membrane was air dried and a grey-scale digital image was made with a transilluminator and saved in TIFF format. The image was then imported to ImageJ (NIMH) software. The image, with 256 grey levels, was enlarged to 135% to facilitate measurement [11].

### 4.5. ELISA Assay

ELISA assay was carried out as previously described. Microtiter flat bottom plates (NUNC Maxi sorp) were coated with 50 µL of LPS O3, (dilution 5 µg/mL) and incubated at 4 °C overnight. The plates were washed three times with phosphate-buffered saline (PBS), blocked with 2% BSA (Sigma-Aldrich) at 37 °C for 2 h, and then washed again three times with PBS. Then, 100 µL of sera sample diluted in 1:1000 (*v*/*v*) in PBS with 1% BSA was added to the wells. After 1h of incubation at 37 °C with shaking, plates were washed five times with PBS+Tween and reacted with 50 µL of secondary antibodies (goat anti-human IgG or IgM, conjugated with horseradish peroxidase (Sigma-Aldrich) diluted 1:1000 (*v*/*v*) in PBS with 1% BSA. Subsequently, plates were washed three times with PBS. In the last stage, 50 µL of SIGMAFAST OPD (Sigma-Aldrich) was added. Plates were incubated in the dark for 20 min then stop reaction by 3M H_2_SO_4_. The absorbance was measured at 492 nm using a multimode microplate reader (Tecan Infinity 2000), as described previously [22]. 

### 4.6. RF Detection

The presence of RF and the approximate concentration was measured using the slide latex agglutination test (Biosystems, Barcelona, Spain) according to the manufacturer’s instructions.

### 4.7. ACPA Detection

The concentration of ACPA was measured by the Anti-citrullinated protein antibodies enzyme immunoassay microplate test (Biosystems, Barcelona, Spain) according to the manufacturer’s instructions.

### 4.8. Measurement of IR Spectra of Sera and Spectroscopic Data Processing

Sera from healthy blood donors and RA patients were frozen at −20 °C. Before measurement, the sera were allowed to thaw at room temperature. Directly before application to the spectroscope ATR crystal, sera were intensively vortexed for a few seconds. A small volume of serum (0.5 μL) was placed on the ATR crystal of the spectroscope. After 5 min, the sample was evaporated, leaving a precipitate on the crystal. The IR spectrum of the precipitate was measured in the range of 4000–750 cm^−1^. Pre-treatment of spectroscopic data included: averaging spectra from three replicates, aligning the baseline, and calculating the first derivative of absorbance.

### 4.9. Antibody Adsorption Assay

Microtiter flat bottom plates (NUNC Maxi sorp, Thermo Fisher, Waltham, MA, USA) were coated with 50 µL of synthetic antigens: Lys-GalA, Thr-GalA, Glc (dilution 5 µg/mL)-adsorbing plates, and with 50 µL LPS O3 (dilution 5 µg/mL) test plates, and incubated at 4 °C overnight. The plates were washed three times with phosphate-buffered saline (PBS), blocked with 2% BSA (Sigma-Aldrich) at 37 °C for 2 h, and then washed again three times with PBS. Then, 100 µL of sera sample diluted in 1:1000 (*v*/*v*) in PBS with 1% BSA was added to the wells of the adsorbing plate in triplicate. After 30 min of incubation at 37 °C with shaking, 50 µL of serum was transferred to test plates and incubated at 37 °C for 1 h. Next steps were performed as ELISA assay describes above [22].

### 4.10. Affinity Purification of Human Anti-LPS O3 Antibodies

Purified *P. mirabilis* O3 lipopolysaccharide was suspended in 10% DMSO in PBS (pH = 7.4) and then coupled to epoxy-activated Sepharose 6B (GE Healthcare, Uppsala, Sweden) through the epoxy linkage according to the manufacturer’s instructions. An amount of 3 mL of serum diluted in 20 mL phosphate-buffered saline (PBS) was loaded onto a column and allowed to bind to LPS for 2 h in 37 °C. After PBS washing, the bound antibodies were eluted from the affinity gel with 0.5 M acetic acid with PBS, and the pH was immediately adjusted with 1 M Tris. Obtained antibodies were dialyzed against PBS. The concentration of antibodies was measured using a spectrophotometer (NanoDrop 2000, Thermo fhisher, Wilmington, Delaware USA) at 280 nm.

### 4.11. Statistical Analysis

All parameters (except age) were distributed non-normally. Comparisons between groups were analyzed using Mann–Whitney U test. Correlations between variables were completed using Spearman’s rank correlation. The conventional statistical significance criterion of α = 0.05 was used. The chi-square test was used to detect wavenumbers related to the sera reactivity with LPS. All analyses were performed using STATISTICA 13.1 software (U.S.A).

## References

[1] Ramos P.S. (2017). Population genetics and natural selection in rheumatic disease Paula. Rheum. Dis. Clin. N. Am..

[2] Pretorius E., Akeredolu O.O., Soma P., Kell D.B. (2017). Major involvement of bacterial components in rheumatoid arthritis and its accompanying oxidative stress, systemic inflammation and hypercoagulability. Exp. Biol. Med..

[3] Lorenz W., Buhrmann C., Mobasheri A., Lueders C., Shakibaei M. (2013). Bacterial lipopolysaccharides form procollagen-endotoxin complexes that trigger cartilage inflammation and degeneration: Implications for the development of rheumatoid arthritis. Arthritis Res. Ther..

[4] Li S., Yu Y., Yue Y., Zhang Z., Su K. (2013). Microbial Infection and Rheumatoid Arthritis. J. Clin. Cell. Immunol..

[5] Terato K., Waritani T., Fukai R., Shionoya H., Itoh H., Katayama K. (2018). Contribution of bacterial pathogens to evoking serological disease markers and aggravating disease activity in rheumatoid arthritis. PLoS ONE.

[6] Ebringer A., Rashid T., Wilson C. (2010). Rheumatoid arthritis, *Proteus*, anti-CCP antibodies and Karl Popper. Autoimmun. Rev..

[7] Deighton C.M., Gray J., Bint A.J., Walker D.J. (1992). Specificity of the *Proteus* antibody response in rheumatoid arthritis. Ann. Rheum. Dis..

[8] Christopoulos G., Christopoulou V., Routsias J.G., Babionitakis A., Antoniadis C., Vaiopoulos G. (2017). Greek rheumatoid arthritis patients have elevated levels of antibodies against antigens from *Proteus mirabilis*. Clin. Rheumatol..

[9] Ebringer A., Rashid T. (2006). Rheumatoid arthritis is an autoimmune disease triggered by *Proteus* urinary tract infection. Clin. Dev. Immunol..

[10] Konieczna I., Kwinkowski M., Kolesinska B., Kaminski Z., Zarnowiec P., Kaca W. (2012). Detection of Antibodies Against Synthetic Peptides Mimicking Ureases Fragments in Sera of Rheumatoid Arthritis Patients. Protein Pept. Lett..

[11] Konieczna I., Relich I., Durajski M., Lechowicz L., Chrapek M., Gaweda J., Fraczyk J., Kaminski Z.J. (2018). Novel tool in rheumatoid arthritis diagnosis—The usage of urease flap region peptidomimetics. J. Pept. Sci..

[12] Różalski A., Kwil I., Torzewska A., Baranowska M., Stączek P. (2007). Bakterie z rodzaju *Proteus*—Cechy i czynniki chorobotwórczości *Proteus* bacilli: Features and virulence factors. Postep. Hig. Med. Dosw..

[13] Kell D.B., Pretorius E. (2015). On the translocation of bacteria and their lipopolysaccharides between blood and peripheral locations in chronic, inflammatory diseases: The central roles of LPS and LPS-induced cell death. Integr. Biol..

[14] Poolman T.M., Gibbs J., Walker A.L., Dickson S., Farrell L., Hensman J., Kendall A.C., Maidstone R., Warwood S., Loudon A. (2019). Rheumatoid arthritis reprograms circadian output pathways. Arthritis Res. Ther..

[15] Jin X.N., Yan E.Z., Wang H.M., Sui H.J., Liu Z., Gao W., Jin Y. (2016). Hyperoside exerts anti-inflammatory and anti-arthritic effects in LPS-stimulated human fibroblast-like synoviocytes in vitro and in mice with collagen-induced arthritis. Acta Pharmacol. Sin..

[16] Grover R.K., Cheng J., Peng Y., Jones T.M., Ruiz D.I., Ulevitch R.J., Glass J.I., Dennis E.A., Salomon D.R., Lerner R.A. (2012). The costimulatory immunogen LPS induces the B-cell clones that infiltrate transplanted human kidneys. Proc. Natl. Acad. Sci. USA.

[17] Knirel Y.A., Perepelov A.V., Kondakova A.N., Senchenkova S.N., Sidorczyk Z., Rozalski A., Kaca W. (2010). Structure and serology of O-antigens as the basis for classification of *Proteus* strains. Innate Immun..

[18] Siwińska M., Levina E.A., Ovchinnikova O.G., Drzewiecka D., Shashkov A.S., Rózalski A., Knirel Y.A. (2015). Classification of a *Proteus* penneri clinical isolate with a unique O-antigen structure to a new *Proteus* serogroup, O80. Carbohydr. Res..

[19] Larsson P. (1984). 6 Serology of *Proteus mirabilis* and *Proteus vulgaris*. Methods Microbiol..

[20] Palusiak A., Maciejewska A., Ługowski C., Rózalski A. (2014). The amide of galacturonic acid with lysine as an immunodominant component of the lipopolysaccharide core region from *Proteus penneri* 42 strain. Acta Biochim. Pol..

[21] Sidorczyk Z., Zych K., Toukach F.V., Arbatsky N.P., Zablotni A., Shashkov A.S., Knirel Y.A. (2002). Structure of the O-polysaccharide and classification of *Proteus mirabilis* strain G1 in *Proteus* serogroup O3. Eur. J. Biochem..

[22] Gleńska-Olender J., Durlik K., Konieczna I., Kowalska P., Gawęda J., Kaca W. (2017). Detection of human antibodies binding with smooth and rough LPSs from *Proteus mirabilis* O3 strains S1959, R110, R45. Antonie van Leeuwenhoek. Int. J. Gen. Mol. Microbiol..

[23] Maijer K.I., Gudmann N.S., Karsdal M.A., Gerlag D.M., Tak P.P., Bay-Jensen A.C. (2016). Neo-epitopes-fragments of cartilage and connective tissue degradation in early rheumatoid arthritis and unclassified arthritis. PLoS ONE.

[24] Doi T., Higashino K., Kuriharag Y., Wadat Y., Miyazakij T., Nakamurali H., Uesugi S., Imanishis T., Itakura H., Yazakij Y. (1993). Charged Collagen Structure Mediates the Recognition of Negatively Charged Macromolecules by Macrophage Scavenger Receptors. Biol. Chem..

[25] Terajima M., Perdivara I., Sricholpech M., Deguchi Y., Pleshko N., Tomer K.B., Yamauchi M. (2014). Glycosylation and cross-linking in bone type I collagen. J. Biol. Chem..

[26] Rashid T., Ebringer A. (2012). Autoimmunity in rheumatic diseases is induced by microbial infections via crossreactivity or molecular mimicry. Autoimmune Dis..

[27] Arabski M., Fudala R., Koza A., Wasik S., Futoma-Koloch B., Bugla-Ploskonska G., Kaca W. (2012). The presence of anti-LPS antibodies and human serum activity against *Proteus mirabilis* S/R forms in correlation with TLR4 (Thr399Ile) gene polymorphism in rheumatoid arthritis. Clin. Biochem..

[28] Prince H.E., Yeh C., Lapé-Nixon M. (2011). Utility of IgM/IgG Ratio and IgG Avidity for Distinguishing Primary and Secondary Dengue Virus Infections Using Sera Collected More than 30 Days after Disease Onset. Clin. Vaccine Immunol..

[29] Haji-Ghassemi O., Müller-Loennies S., Rodriguez T., Brade L., Kosma P., Brade H., Evans S.V. (2015). Structural basis for antibody recognition of lipid A: Insights to polyspecificity toward single-stranded DNA. J. Biol. Chem..

[30] Helmerhorst E.J., Maaskant J.J., Appelmelk B.J. (1998). Anti-lipid A monoclonal antibody Centoxin (HA-1A) binds to a wide variety of hydrophobic ligands. Infect. Immun..

[31] Bhat N.M., Bieber M.M., Chapman C.J., Stevenson F.K., Teng N.N.H. (1993). Human antilipid A monoclonal antibodies bind to human B cells and the i antigen on cord red blood cells. J. Immunol..

[32] Radziejewska-Lebrecht J., Shashkov A.S., Vinogradov E.V., Grosskurth H., Bartodziejska B., Rozalski A., Kaca W., Kononov L.O., Chernyak A.Y., Mayer H. (1995). Structure and Epitope Characterisation of the O-specific Polysaccharide of *Proteus mirabilis* O28 Containing Amides of d-galacturonic Acid with l-serine and l-lysine. Eur. J. Biochem..

[33] Patel N., Mills P., Davison J., Cleary M., Gissen P., Banushi B., Doykov I., Dorman M., Mills K., Heywood W.E. (2019). Free urinary glycosylated hydroxylysine as an indicator of altered collagen degradation in the mucopolysaccharidoses. J. Inherit. Metab. Dis..

[34] Bäcklund J., Treschow A., Bockermann R., Holm B., Issazadeh-navikas S., Kihlberg J., Holmdahl R. (2002). Glycosylation of type II collagen is of major importance for T cell tolerance and pathology in collagen-induced arthritis. Eur. J. Immunol..

[35] Vinogradov E. (2011). Structure of the core part of the lipopolysaccharide from *Proteus mirabilis* genomic strain HI4320. Biochemistry.

[36] Vinogradov E.V., Krajewska-Pietrasik D., Kaca W., Shashkov A.S., Knirel Y.A., Kochetkov N.K. (1989). Structure of *Proteus mirabilis* 027 O-specific polysaccharide containing amino acids and phosphoethanolamine. Eur. J. Biochem..

[37] Chernyak A.Y., Kononov L.O., Kochetkov N.K. (1994). Glycopolymers from synthetic fragments (amides of α-d-galacturonic acid with amino acids) of *Proteus* o-antigens. J. Carbohydr. Chem..

